# Acute Pleurisy

**Published:** 1861-07

**Authors:** 


II. *Acute Pleurisy: Recovery.*—C. P. J., æt 37, of strong constitution, native of Sweden, was admitted into Marine Hospital, June 5th. No hereditary predisposition to pulmonary disease traceable; has enjoyed good health until the present attack of illness. On the 27th of May, after exposure to wet, he was seized with a chill which lasted for two hours, followed by a fever; these symptoms were succeeded by a cough and a sharp pain, one inch below the left nipple, darting through to the back, rendering him in a short time unable to inhale a free breath. The dyspnœa and local symptoms increased in severity, but the febrile symptoms had somewhat abated on admission. No expectoration with cough. Physical examination—nutrition fair; left chest does not expand upon forcible inspiration, and measures one inch more than right. Vocal vibrations are not felt upon the left chest, below the level of the angle of the scapula; on percussion the right chest is resonant throughout; left chest, laterally, dulness from the fourth rib downward—posteriorly, flatness below the angle of scapula; respiration of right chest, *puerile;* on listening over the dull surface of left chest respiration is entirely wanting, and strongly increased vocal resonance; œgophony is present marked by the sixth rib; no friction sound or crepitating murmur is distinguished; no bronchial inspiration; skin dry; tongue furred; pulse 102, of natural strength; no appetite; bowels constipated; urine scanty, and loaded with urates.

June 7. Dyspnœa and pain much relieved; pulse 90, and feeble; physical signs the same.

June 19. The extent of dulness, œgophony, and vocal resonance sensibly decreased, and respiratory murmurs are faintly heard, but friction sounds plainly distinguished over the extent of surface marked by previous dulness; pulse 76; pain quite gone.

June 20. Friction sounds absent; slight dulness remaining over base of left chest; respiratory murmur heard throughout the lung, and pain entirely absent; patient convalescing; pulse 64; treatment prescribed was the combined use of squills, digitalis, and mercury, and flying blisters to the chest.

The above was a case of uncomplicated pleuritis, with effusion and sufficient exudation of serum to so compress the lung as to destroy the respiratory murmur. The subsequent absorption of this allowed the pleural surfaces to come into contact, developing the friction sound which was heard late in the progress of the case. The diminished action of the left lung was counterbalanced by the increased action of the right, as evidenced by the puerile respiration.

